# Effectiveness of fluorescence-guided methods using near-infrared fluorescent clips of robotic colorectal surgery: a case report

**DOI:** 10.1186/s40792-023-01666-z

**Published:** 2023-05-17

**Authors:** Satoshi Narihiro, Syunsuke Nakashima, Mutsumi Kazi, Satoshi Yoshioka, Kazuo Kitagawa, Naoki Toya, Ken Eto

**Affiliations:** 1 Department of Surgery, The Jikei University Kashiwa Hospital, 163-1 Kashiwashita, Kashiwa, Chiba 277-8567 Japan; 2 Department of Surgery, The Jikei University School of Medicine, 3-19-18 Nishi-Shinbashi, Minato-Ku, Tokyo, 105-8471 Japan

**Keywords:** Colorectal cancer, Fluorescence-guided methods, Robotic surgery, Near-infrared fluorescent clips

## Abstract

**Background:**

This is the first report on the application of the Da Vinci-compatible near-infrared fluorescent clips (NIRFCs) as tumor markers to localize colorectal cancer lesions during robotic surgery. In laparoscopic and robotic colorectal surgeries, the accuracy of tumor marking is a critical issue that remains unresolved. This study aimed to determine the accuracy of NIRFCs in localizing tumors for intestinal resection. Indocyanine green (ICG) was also used to verify the feasibility of safely performing an anastomosis.

**Case presentation:**

A patient diagnosed with rectal cancer was scheduled to undergo a robot-assisted high anterior resection. During colonoscopy 1 day prior to the surgery, four Da Vinci-compatible NIRFCs were placed intraluminally 90° around the lesion. The locations of the Da Vinci-compatible NIRFCs were confirmed using firefly technology, and ICG staining was performed before cutting the oral side of the tumor. The locations of the Da Vinci-compatible NIRFCs and the intestinal resection line were confirmed. Moreover, sufficient margins were obtained.

**Conclusions:**

In robotic colorectal surgery, fluorescence guidance with firefly technology offers two advantages. First, it has an oncological advantage, because marking with the Da Vinci-compatible NIRFCs allows for real-time monitoring of the lesion location. This enables sufficient intestinal resection by grasping the lesion precisely. Second, it reduces the risk of postoperative complications, because ICG evaluation with firefly technology prevents postoperative anastomotic leakage. Fluorescence guidance in robot-assisted surgery is useful. In the future, the application of this technique should be evaluated for lower rectal cancer.

## Background

The Da Vinci® Xi surgical system is integrated with fluorescence imaging (firefly technology). The endoscope’s camera contains an infrared excitation laser (805 nm) that visualizes infrared light (830 nm) [1]. The firefly technology enables fluorescence-guided surgery [2, 3].

In laparoscopic and robotic colorectal surgeries, the accuracy of tumor marking is a critical issue that remains unresolved. The tattoo marking technique and intraoperative endoscopy have been used. However, they are associated with an increased risk of accidental intestinal puncture, longer operation time, requirement of a skilled endoscopist, and intraoperative colon insufflation.

Using near-infrared fluorescent clips (NIRFC) for the intraoperative localization of gastrointestinal tumors addresses the disadvantages of tattoo marking and intraoperative endoscopy techniques [4]. The Da Vinci-compatible NIRFC: ZEOCLIP FS® (Zeon Medical, Tokyo, Japan) is a newly designed tumor marking tool in robotic surgery [5].

Since the ideal length of the resection margin (RM) is essential in eliminating lymph node metastasis in the mesentery, using NIFRCs is advantageous, because it accurately localizes the lesions [6]. In contrast, the scattered ink, used in the conventional preoperative tumor site marking methods, affects recognition of the tumor site, leading to inaccurate dissection margins.

Anastomotic leakage (AL) is one of the most critical complications of colorectal surgery. According to a recent study, fluorescence imaging with indocyanine green (ICG) decreased the AL rates [7]. Firefly technology also provided real-time identification of intestinal blood flow using ICG.

This study presented the first case of fluorescent-guided robotic surgery that utilized firefly technology. Da Vinci-compatible NIRFCs were used to localize the rectal cancer lesion. This study also evaluated its ability to determine the intestinal resection length. The intestinal blood flow evaluation was combined with ICG to verify the feasibility of performing an anastomosis.

## Case presentation

A 73-year-old woman with anemia presented to our institution. Based on the lower intestinal endoscopy findings, she was diagnosed with rectal cancer (Rs type 2, 40 mm in size, cT3, cN0, cM0, stage IIa). Subsequently, the patient was scheduled for laparoscopic robot-assisted high anterior resection with D3 lymph node dissection. The patient consented to the intraoperative use of the newly designed fluorescent clip and the publication of the study results. This study was also approved by the Institutional Review Board [No. 30-249(9270)].

**Fig. 1:**
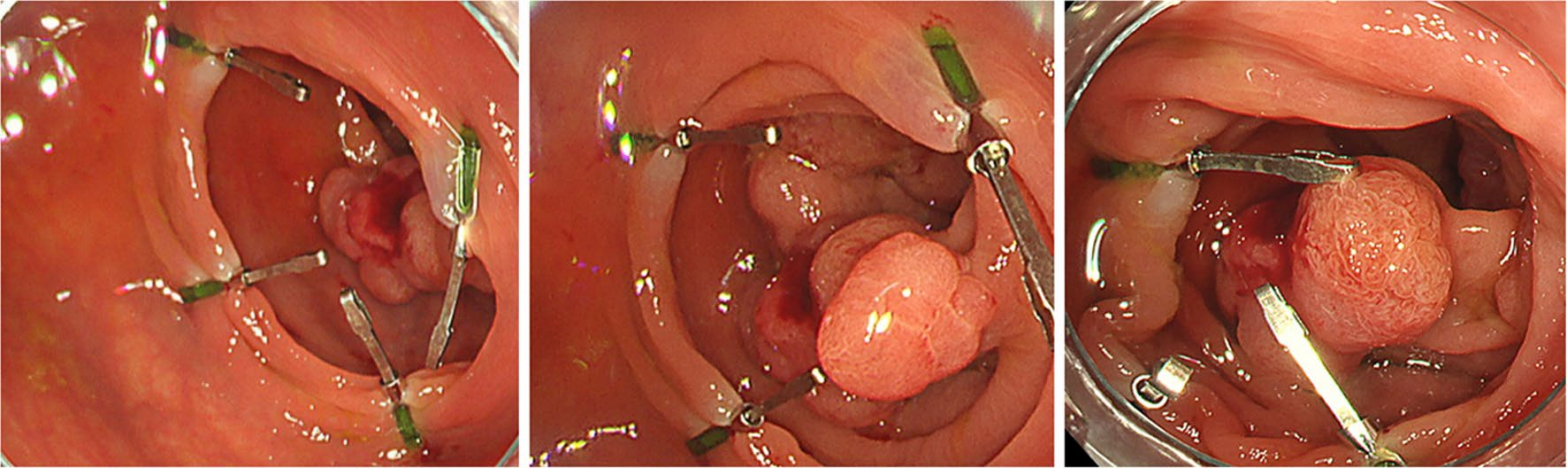
Four clips placed intraluminally 90° around the lesion. The intensity of the near-infrared fluorescent clip (NIRFCs) was sufficient

**Fig. 2:**
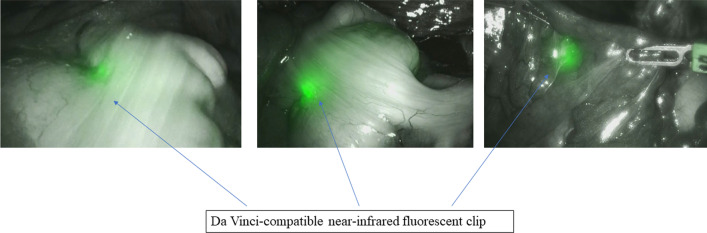
Locations of the Da Vinci-compatible NIRFCs were easily confirmed using firefly technology

**Fig. 3:**
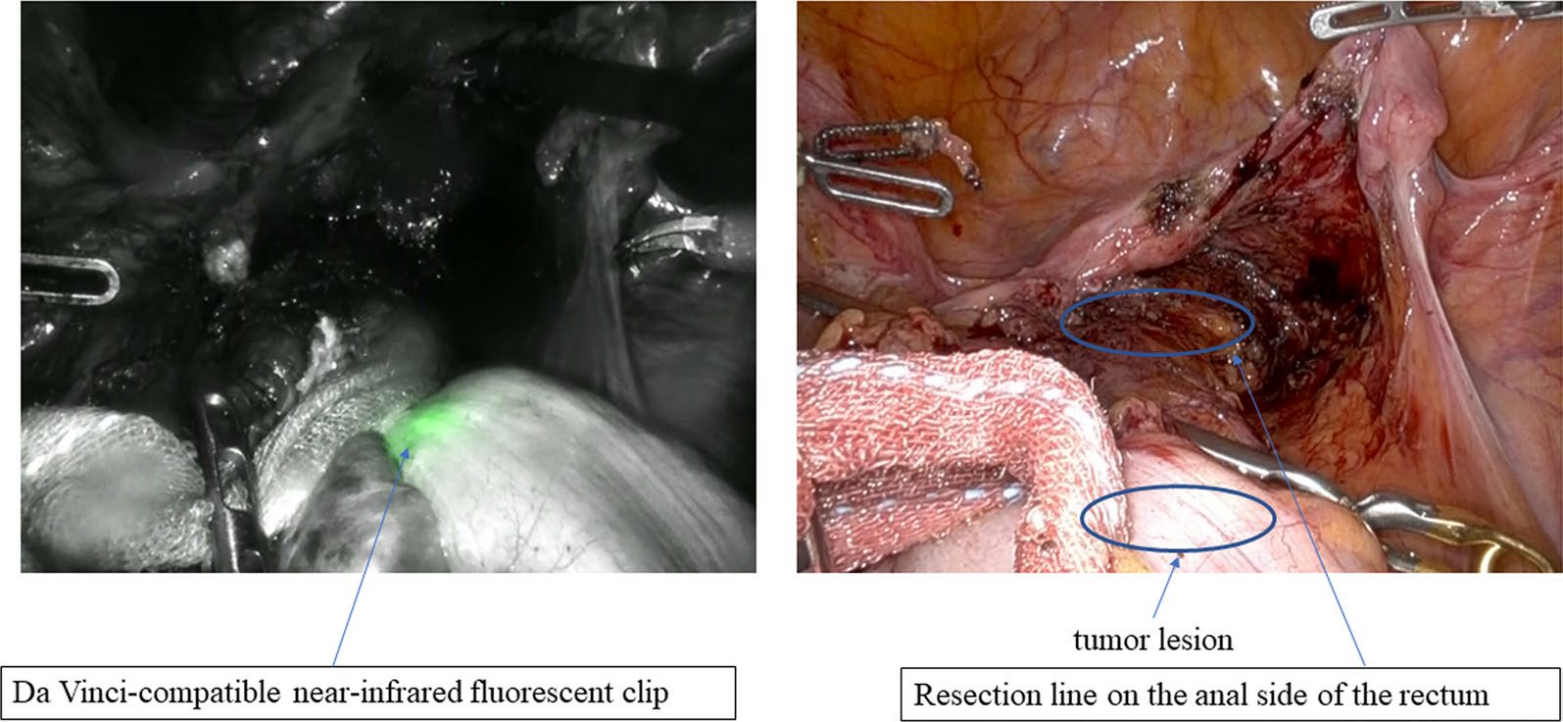
Image showing the real-time intraoperative view and fluorescence imaging view before resection of the anal side of the rectum

**Fig. 4:**
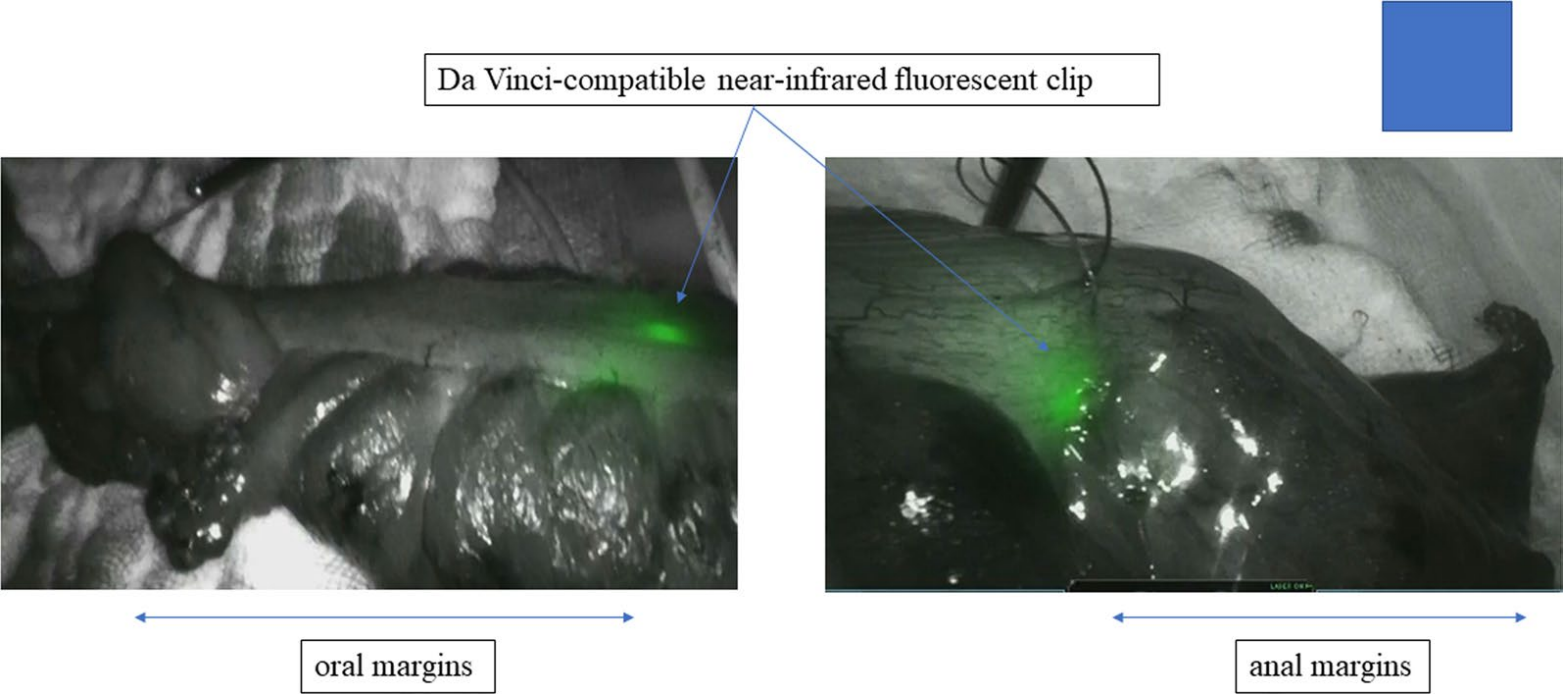
Da Vinci-compatible NIRFCs allowed accurate tumor localization and measurement of the anal and oral margins

**Fig. 5:**
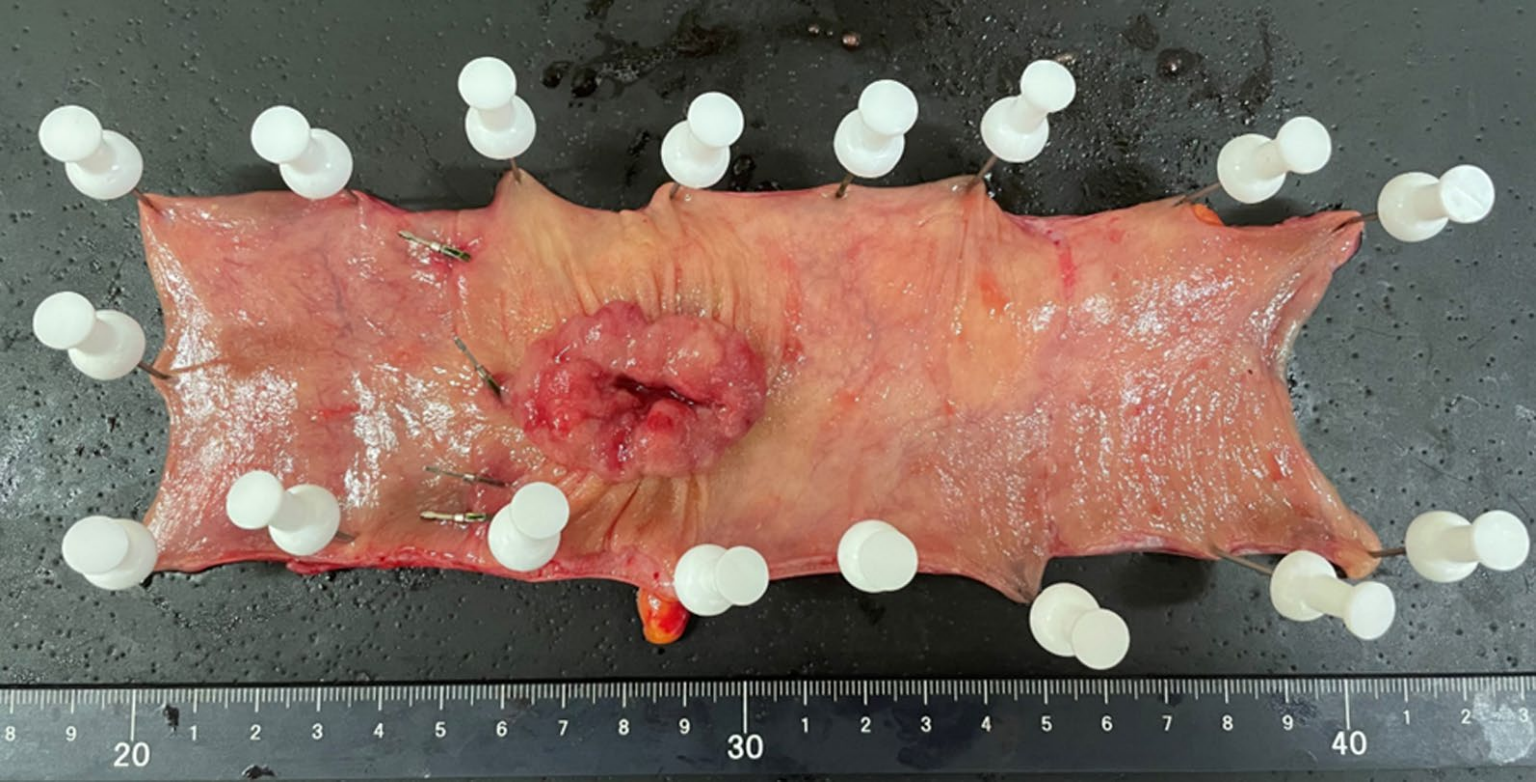
Pathological findings were Rs type 2, 44 × 28 mm in size, pT3, pN0, pM0, pPM0 (100 mm), and pDM0 (55 mm). Four clips were found in each placement location

Following a 2-day bowel preparation, the Da Vinci-compatible NIRFCs were placed during a colonoscopy. Four clips were placed intraluminally 90° around the lesion (Fig. 1). The surgery was performed the following day. The curative operation was performed based on the preoperative radiological results. The locations of the da Vinci-compatible NIRFCs were confirmed using firefly technology (Fig. 2). Sufficient margins were obtained while separating the anal side of the tumor using an automatic suture device (Fig. 3). ICG intestinal blood flow evaluation was performed before cutting the oral side of the tumor. The locations of the Da Vinci-compatible NIRFCs and the intestinal resection line were confirmed. Moreover, sufficient oral and anal margins were obtained (Fig. 4). The operation time was 292 min, and the blood loss volume was 5 mL. No intraoperative complications were observed. Pathological examination confirmed the preoperative findings of Rs type 2 tumor, measuring 44 × 28 mm, pT3, pN0, pM0, pPM0 (100 mm), and pDM0 (55 mm) (Fig. 4). The postoperative course was uneventful, and the patient was discharged on the seventh postoperative day. One year postoperatively, the patient underwent a follow-up evaluation, and no recurrence was detected (Fig. 5).

## Discussion

In robotic colorectal surgery, fluorescent guidance with firefly technology offers two advantages. First, it is advantageous, because the Da Vinci-compatible NIRFCs allow accurate, real-time monitoring of lesion location and sufficient intestinal resection by grasping the lesion precisely. The ideal length of the distal RM (DRM) is an essential factor that regulates the elimination of lymph node metastasis in the mesentery and distal intramural spread in the intestinal wall [6, 8]. Especially, in rectal cancer cases like the present case, a shorter DRM increases the risk of local recurrence and decreases the overall survival rate [9, 10]. Thus, the tumor should be localized accurately. Unlike the conventional tattoo marking method, the Da Vinci-compatible NIRFC does not result in ink scattering. Moreover, the firefly technology makes localizing the tumor easier. The Da Vinci-compatible NIRFC is useful in the management of rectal cancer cases.

Second, it reduces the risk of postoperative complications. ICG evaluation with firefly technology prevents postoperative AL, a severe complication of colorectal cancer. AL occurs in 1–20% of patients. It has been associated with less favorable short-term outcomes, such as a high reoperation rate and prolonged hospital stay, as well as long-term outcomes, such as a high local recurrence rate and low concurrent cancer-specific survival [11]. In laparoscopic surgery, a dedicated ICG camera is used. The number of facilities capable of performing ICG evaluation remains limited. However, the demand for ICG increased in 2018 when it became covered by insurance. The camera, used in the Da Vinci surgery, resulted in an easy ICG evaluation and safe anastomosis [12]. Based on these advantages, robotic surgery can be viewed in real-time.

**Fig. 6:**
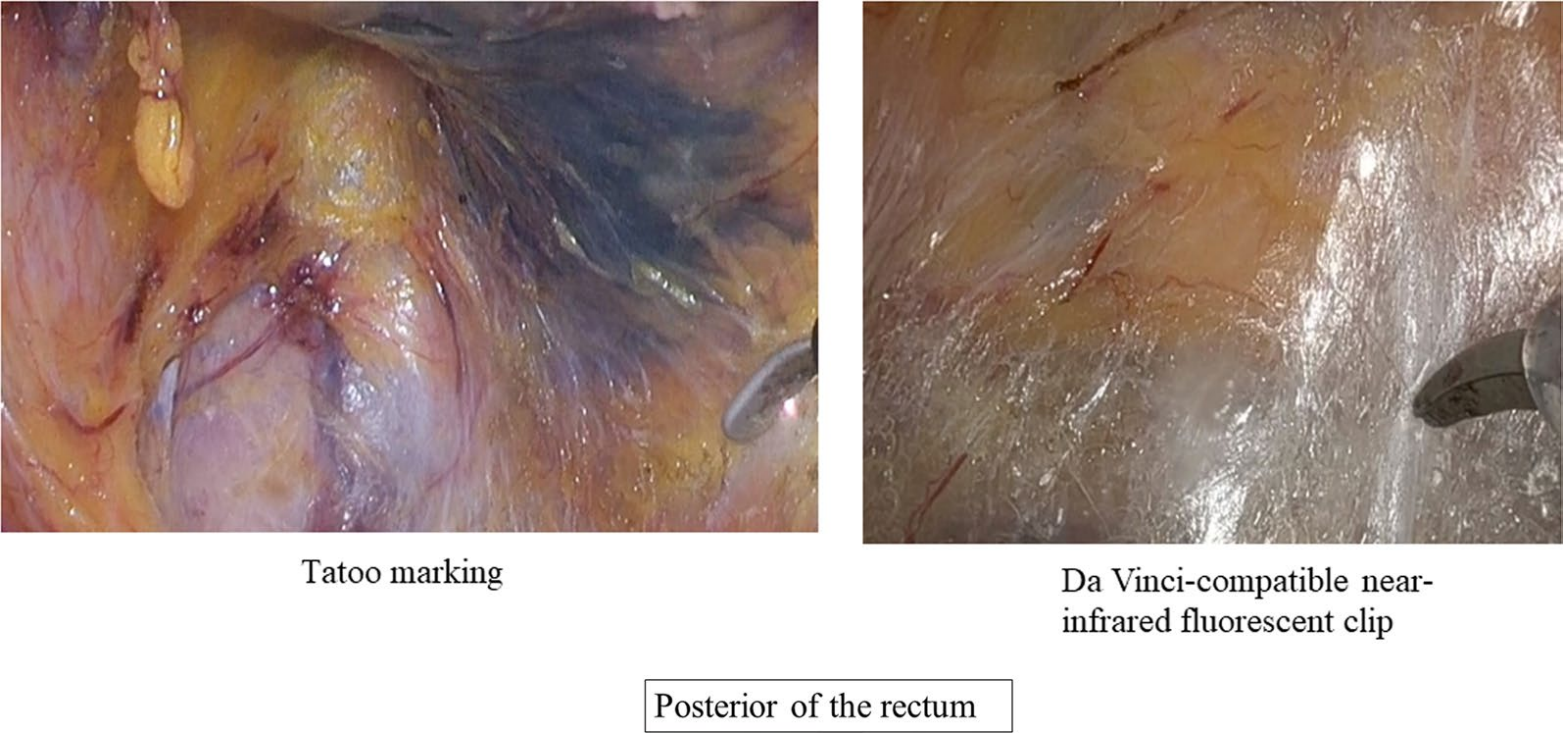
Scattered ink makes it difficult to distinguish the TME layers

The optimal placement, intensity, and clarity for the Da Vinci-compatible NIRFCs will be reviewed by gathering-related cases. In the future, the application of this method in the management of patients with lower rectal cancer should be investigated. In lower rectal cancer, the circumferential RM (CRM) influences the local recurrence rate, like the DRM. After preoperative treatment, the 5-year local recurrence rate for a CRM measuring > 1 mm was significantly lower than those ≤ 1 mm [13]. Total mesorectal excision (TME) surgery is the standard treatment for patients with lower rectal cancer [14]. To achieve a complete TME and ensure a sufficient CRM, tumor site marking with NIRFC may be used instead of tattoo marking, which results in ink scattering. The scattered ink during tattoo marking makes it difficult to recognize the TME layers (Fig. 6). Thus, it is not suitable for patients undergoing TME. The NIRFC*s* marking method is useful for cases of lower rectal cancer.

Aside from TME, chemoradiation therapy and total neoadjuvant therapy are also standard treatment options for patients with lower rectal cancer [14]. These modalities reduce the size of the primary lesion. Consequently, the intestinal resection length is shortened, and the anal function is preserved. Tumor site marking with NIRFC allows a more accurate localization of the lesion.

## Conclusions

The fluorescence-guided method with NIRFC in robotic colorectal surgery provided a more accurate localization of the tumor and reduced the risk of postoperative complications.

## Data Availability

The data sets, supporting the conclusions of this article, were included in the article and its additional files.

## Abbreviations

AL: Anastomotic leakage
CRM: Circumferential resection margin
DRM: Distal resection margin
ICG: Indocyanine green
NIRFC: Near-infrared fluorescent clip
RM: Resection margin
TME: Total mesorectal excision
